# Object Detection of Flexible Objects with Arbitrary Orientation Based on Rotation-Adaptive YOLOv5 †

**DOI:** 10.3390/s23104925

**Published:** 2023-05-20

**Authors:** Jiajun Wu, Lumei Su, Zhiwei Lin, Yuhan Chen, Jiaming Ji, Tianyou Li

**Affiliations:** 1 College of Electrical Engineering and Automation, Xiamen University of Technology, Xiamen 361024, China; 2 Xiamen Key Laboratory of Frontier Electric Power Equipment and Intelligent Control, Xiamen 361024, China

**Keywords:** power grid maintenance and inspection site, object detection, flexible objects with arbitrary orientation, YOLOv5

## Abstract

It is challenging to accurately detect flexible objects with arbitrary orientation from monitoring images in power grid maintenance and inspection sites. This is because these images exhibit a significant imbalance between the foreground and background, which can lead to low detection accuracy when using a horizontal bounding box (HBB) as the detector in general object detection algorithms. Existing multi-oriented detection algorithms that use irregular polygons as the detector can improve accuracy to some extent, but their accuracy is limited due to boundary problems during the training process. This paper proposes a rotation-adaptive YOLOv5 (R_YOLOv5) with a rotated bounding box (RBB) to detect flexible objects with arbitrary orientation, effectively addressing the above issues and achieving high accuracy. Firstly, a long-side representation method is used to add the degree of freedom (DOF) for bounding boxes, enabling accurate detection of flexible objects with large spans, deformable shapes, and small foreground-to-background ratios. Furthermore, the further boundary problem induced by the proposed bounding box strategy is overcome by using classification discretization and symmetric function mapping methods. Finally, the loss function is optimized to ensure training convergence for the new bounding box. To meet various practical requirements, we propose four models with different scales based on YOLOv5, namely R_YOLOv5s, R_YOLOv5m, R_YOLOv5l, and R_YOLOv5x. Experimental results demonstrate that these four models achieve mean average precision (mAP) values of 0.712, 0.731, 0.736, and 0.745 on the DOTA-v1.5 dataset and 0.579, 0.629, 0.689, and 0.713 on our self-built FO dataset, exhibiting higher recognition accuracy and a stronger generalization ability. Among them, R_YOLOv5x achieves a mAP that is about 6.84% higher than ReDet on the DOTAv-1.5 dataset and at least 2% higher than the original YOLOv5 model on the FO dataset.

## 1. Introduction

Object detection is one of the most difficult tasks in computer vision, as it involves classifying and locating objects from an input image. Object detection based on deep learning has been widely used in various fields, such as pose estimation [1], underwater object detection [2], and aerial image detection [3], due to its high accuracy, generality for different objects, and transferability. Most of those based on deep learning [4,5,6,7,8,9,10,11] apply a horizontal regression method to locate the horizontal bounding box (HBB) that describes the spatial position of the objects using a rectangular boundary. Although this method is applicable to most cases, it is difficult to use for detecting flexible objects with significant morphological variation.

Flexible objects in images typically refer to objects with irregular shapes and large spans. In power plants, mines, and other workplaces, it is significant for worker safety to accurately detect flexible objects such as safety fences and area-dividing isolation belts in safety monitoring videos. General object detection algorithms commonly utilize the HBB as the detector, and the detection box produced by these algorithms lacks angle information, leading to redundant background information in the detection box and lower detection efficiency when detecting flexible objects. The rotated bounding box (RBB) [12] introduces angle parameters to extract directional features of flexible objects, which greatly improves the detection efficiency of such objects. However, the angle parameters used in this method are limited by their value range, which may lead to redundant parameter regression during the training process, resulting in limited detection accuracy.

On the problem of detecting flexible objects with arbitrary orientations, Chen et al. [13] proposed a method for segmenting slender flexible objects by adding an object correlation module and optimizing the loss function, but object segmentation requires more complex models and more computational resources to obtain high-quality segmentation results, which also leads to a decrease in inference speed. Kong et al. [14] used a method based on keypoint-displacement patterns to detect uncertain elongated objects; however, this method requires defining a relatively large area around each key point to overcome the uncertainty of object orientation and scale, which leads to significant computational and spatial costs. Wan et al. [15] proposed a feature-adaptive strategy for detecting elongated objects after identifying the key factors for detecting such objects; however, the performance of this method is also affected by factors such as data quality, model architecture, and the choice of hyperparameters, which may require more computational resources for training and inference. Jiang et al. [16] applied a double-shot neural network and misplaced localization strategy to the detection network to achieve better positioning of small, narrow, and differently oriented objects in high-resolution satellite images (HRSI). This method requires a more complex network architecture and more computing resources and requires displacement positioning for each object, which will reduce the model’s inference speed. The above method cannot detect flexible objects in real time, so the safety of personnel in the power scene cannot be guaranteed. Moreover, existing research on flexible object detection with arbitrary orientations has mainly focused on improving feature extraction performance, detection accuracy, and training speed [4,5,6,7,8,9,10,11], while few studies focus on object directional information and bounding box accuracy.

**Figure 1:**
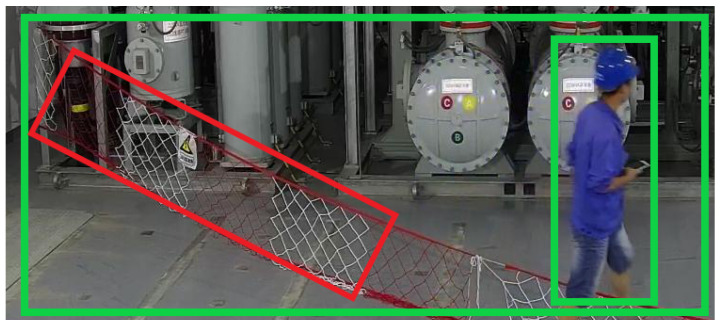
Comparison of the actual scene detection result between HBB and RBB.

In this discussion, we are concerned with the bounding box’s accuracy. Taking Figure 1 as an example, the green bounding box is the ideal result of labeling the seine and people with the HBB, and the red bounding box is the ideal result of labeling the seine with the RBB. The objects with narrow spans are accurately detected by using the HBB to identify and locate objects, such as the human in Figure 1. However, it is not suitable for detecting objects with large spanning patterns, such as the seine. For such objects, the HBB will include more backgrounds, as shown in the largest green rectangular box in Figure 1. On the contrary, using an RBB can accurately locate the seine, as shown in the red box in Figure 1. The RBB is suitable to be used as a detector to detect flexible objects with arbitrary orientation, such as the seine and fence.

The contributions summarized in this paper are as follows: (1) The parameters for learnable angles are added to the YOLO head so that the model can learn the object’s orientation information. (2) To ensure that the anchor does not exceed the range when returning to the ground truth (GT) angle, the classification approach is proposed to solve the numerical regression problem. (3) A symmetric function restriction is added to solve the periodic problem of the angle parameters while simultaneously mapping the numerical distance between them to match the loss function’s calculation requirements. This is a new rotation-adaptive YOLOv5 object detection method suitable for detecting flexible objects with arbitrary orientation in power monitoring scenarios.

In this paper, we first briefly review the challenges and practices of existing object detection algorithms and detectors in detecting flexible objects with arbitrary orientation in Section 2 and then discuss our methods to address these problems.

## 2. Related Work

Most object detection algorithms fail to detect flexible objects that are arbitrarily oriented with high detection precision due to the omission of learning directional features. Thus, it is of great research significance to improve the original detector for the accurate detection of such objects. The following is a discussion of existing works aimed at improving the detection accuracy and bounding box accuracy of objects based on deep general object detection algorithms and object detectors with different shapes.

### 2.1. Deep General Object Detection

Object detection works based on deep learning and commonly uses the HBB as the detector, and the detection tasks mainly focus on object classification and object localization. Many excellent network models have been proposed for normal object detection. The region-convolutional neural network (R-CNN) [4] was the first proposed convolutional neural network (CNN) model based on an object detection method that uses a selective search algorithm to find prospective regions, extract features by CNN, and finally classify them by the support vector machine (SVM). Although the performance of R-CNN is significantly better than that of previous algorithms, extracting features from each prospective region leads to rather laborious calculations and excessive memory consumption. To solve this problem, Fast R-CNN [6], Faster R-CNN [7], and R-FCN [8] were successively proposed. All of them apply the idea of the pooling pyramid used in SPP-Net [5], which improves detection speed while reducing computational storage. Fast R-CNN [6] simplifies the framework of R-CNN [4], completes localization and classification tasks at the same time, and saves storage space. Based on the former proposed R-CNN models, Faster R-CNN [7] adopts the end-to-end training and testing mode. The structure of feature pyramid networks (FPN) for object detection [9] focuses on tackling the problem of object changes in different proportions, and through the fusion of feature layers, FPN not only captures richer semantic features but also provides multi-scale feature information. SSD [10], YOLO [11], SqueezeDet [17], and DetectNet [18] are the most common one-stage detection algorithms that detect things quickly due to a one-stage structure.

Among them, the YOLO series of deep-learning-based object detection algorithms has gained widespread attention in the academic community due to its excellent speed–accuracy trade-off performance and powerful end-to-end detection capabilities. It has become one of the most widely used visual detection techniques in many applications of object detection, particularly in the HBB detector. YOLOv1 [11] was the first version in the YOLO family, which abandoned the traditional sliding window technique and instead employed a fully convolutional neural network (FCN) to divide the input image into multiple grid cells and predict the object class and location within each grid cell. The YOLOv2 [19] algorithm builds upon the YOLOv1 by introducing a series of improvements, including the use of a simpler network architecture called DarkNet19 [20] and the incorporation of anchor boxes to handle objects of different scales. Inspired by ResNet [21], YOLOv3 [22] further enhances the network depth by introducing residual structures to prevent convergence issues associated with deep networks. Additionally, YOLOv3 innovatively utilizes multi-scale feature maps and a feature pyramid network (FPN) [9] structure, resulting in significant improvements in detection accuracy of the model. YOLOv4 further improves upon YOLOv3 by introducing SPPNet [5], CmBN [23], CSPNet [24], and other techniques to achieve dual enhancements in accuracy and speed. The most significant changes in YOLOv5 compared to previous versions in the YOLO series lie in its adoption of a completely new network architecture, along with a combination of various backbone networks, such as CSPNet [24]; EfficientNet [25]; and Neck networks, such as PANet [26], FPN [9], etc. These networks make YOLOv5 more powerful in terms of feature extraction and information fusion from images.

The difficulty of the accurate detection of flexible objects with arbitrary orientations is increased due to the geometric changes caused by factors such as object scale, altitude, viewpoint, and partial deformation. Some object detection algorithms represented by RepPoints [27] are proposed to detect deformable objects. Deformable convolution operators can extract rich feature information from flexible objects in arbitrary directions, but they also bring heavy computational costs. In addition, if the algorithm still uses the HBB as the detector, the problem of the imbalance between the foreground and background of the detected target still exists.

### 2.2. Arbitrarily Oriented Object Detection

The object detection of arbitrarily oriented objects has not received extensive attention from researchers, so the existing algorithms do not have a good effect on detecting these objects. Instance segmentation is one of the best methods for detecting these objects, but due to the detector’s rectangular shape and slow inference speed, it is not suitable for detecting arbitrarily oriented objects. On the other hand, in the subdivision field of object detection, another way to enhance the feature extraction capability of the network across regions is to apply the detector with other shapes [12,28,29,30,31] to reduce the interference of background information from the label without adding additional computational burden to the network.

The detectors used in existing object detection algorithms can be classified into three types.

**Figure 2:**
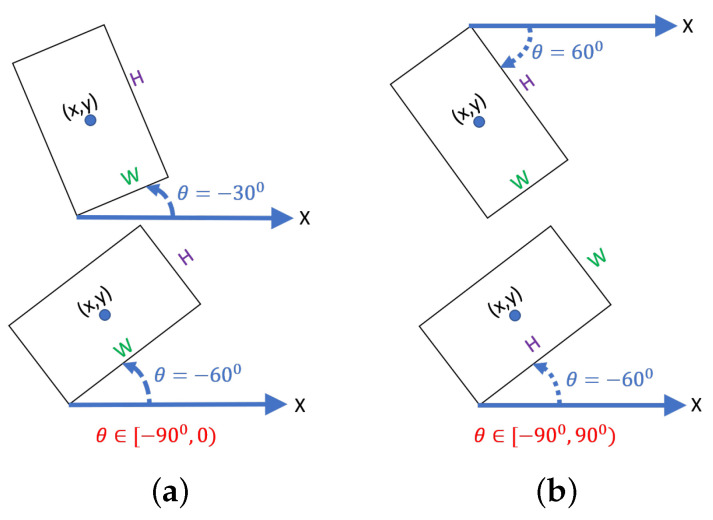
Two popular representations of rotated bounding boxes. (**a**) OpenCV representation. (**b**) Long-side representation.

(1) Horizontal bounding boxes: The HBB is the detector of the current mainstream object detection algorithm [4,5,6,7,8,9,10,11]. When the scale and shape of the foreground object do not greatly change, the HBB can accurately represent the object’s position and shape.

(2) Rotated bounding boxes: This type of model extracts the directional features of the object by increasing the degrees of freedom of the HBB [12,28,29,30,31]. The detection of the RBB is compatible with that of the HBB, and the detection accuracy of the RBB on flexible objects with arbitrary orientation is better than that of the HBB. The OpenCV representation and Long-side representation method are two common representations for the RBB, as shown in Figure 2. However, each has the problem of representing the boundary.

The notation of OpenCV and long-side representation is [x,y,W,H,θ]. The meaning of the parameters is as follows: the *x* and *y* coordinate offsets of the center of the rectangular box, the width and height of the rectangular box, and the rotation angle of the rectangular box are used to describe the rotatable bounding box. The angle θ of the OpenCV representation refers to the acute angle formed by the bounding box and the x-axis. The side that forms an acute angle between the bounding box and the x-axis is marked as *W*, and the other side is marked as *H*; the range of the angle is [$-90^{\circ },0^{\circ }$). The angle θ of the long-side representation refers to the angle formed by the longest side of the bounding box and the x-axis; the range of the angle is [$-90^{\circ },90^{\circ }$). The longest side is locked as *H* and the short side as *W*.

**Figure 3:**
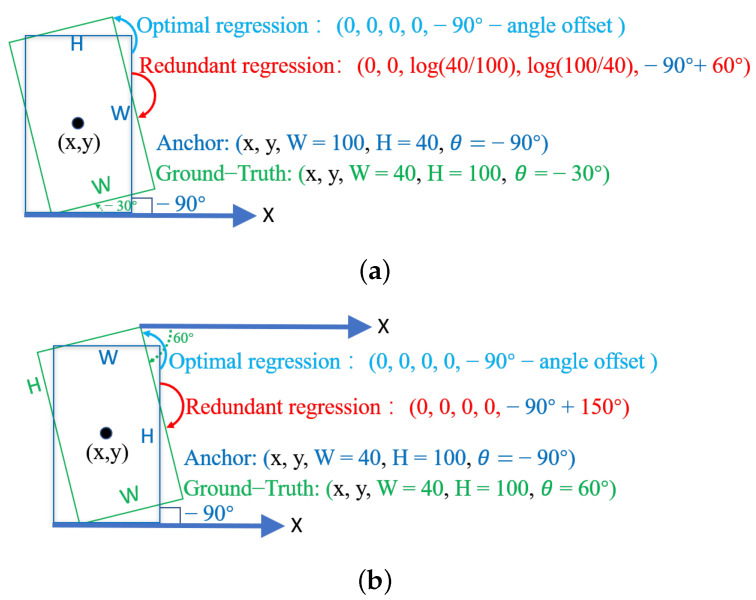
Boundary problems with OpenCV and long-side representation. (**a**) Boundary problems of OpenCV representation. (**b**) Boundary problems of long-side representation.

The details of the boundary problem for the two representations are shown in Figure 3. First, the blue bounding box is the anchor, and the green bounding box is the GT bounding box. The coordinates of the two bounding boxes are shown in the figure, and the coordinates of the center point are consistent. We only analyze the [W,H,θ] parameters of the two bounding boxes. Then, the figure shows the coordinate process of the anchor regression to the GT bounding box. The counterclockwise indigo arrow is the optimal regression path, and the clockwise red arrow is the redundant regression path. Finally, we analyze the boundary problem of the two representations on the optimal regression path and the redundant regression path. On the optimal regression path, both OpenCV and the long-side representation have an angle out-of-bounds problem when moving counterclockwise. Specifically, when the anchor suggestion box performs the $-90^{\circ }-angleoffset$ numerical operation and rotates counterclockwise, it will exceed the angle range of the two representations. On the redundant regression path, the OpenCV representation not only has the problem of crossing the boundary of the angle, but the long and short sides of the OpenCV representation are also not locked, and redundant scaling operations are required, that is, the long and short side exchange problem is unique to OpenCV. In contrast, the long and short sides of the long-side representation are locked, and there is only the same angle out-of-bounds problem as the optimal regression path.

(3) Other boundary boxes: Other boundary box methods are proposed by fundamentally changing the shape of detectors. For example, a four-corner point representation method is proposed to detect irregular objects without the shape restriction of a rectangular box. However, determining the labeling criteria and dealing with the best regression priority for the four corner points are required when using the four-corner point representation.

The boundary problem also discussed in CSL [32] arises when using the RBB as a detector. It is built by adding the rotational degrees of freedom of the HBB. After introducing the angle parameter, the regression numerical operation is required, and it probably extends beyond the angle’s limited range, which would inevitably have an impact on training. Moreover, the variation of the angle value is not continuously at the critical point of the angle representation because of the representation method of the RBB. The appearance of a rapid change makes backpropagation of the training impossible.

SCRDet [12], ICN [33], and R3Det [34] are the most representative algorithms for rotating object detection. SCRDet [12] is proposed to detect remote sensing objects. Because the objects shot at high altitudes are small and dense, and the direction is random, the OpenCV representation is applied in SCRDet [12]. Although the IoU-Smooth L1 Loss is optimized in SCRDet [12] to overcome the boundary problem to accurately detect small, dense, and arbitrary objects, suppression does not fix the problem in the long run. Quadrilateral object detection has an optimal regression problem for the corner points of the detection boxes during training. Most object detection methods use the quadrilateral detector to bounding deformed objects without handling timing information well. The gliding vertex [30] first roughly detects the objects by using the HBB and then offsets the four corners of the horizontal rectangular frame to reduce superfluous background information. RSDet [31] presents a sorting algorithm for four corner points to ensure the uniqueness of the corner point sequence.

In general, current object detection algorithms are inadequate for reliably detecting flexible objects. According to the above discussion, we choose to introduce the degree of freedom directly into the HBB in existing deep general object detection methods. Specifically, we add an angle parameter that represents the orientation of the detection box while retaining the position and size parameters of the HBB. This method not only ensures the basic energy efficiency of the object detection algorithm, but also improves the detection accuracy of flexible objects with arbitrary orientation and successfully eliminates the problems of fewer HBBs occupied by foreground objects and a low detection rate.

### 2.3. Discussion of Related Works

Above all, the HBB focuses on identifying the object category and positioning offset of a possible region, and it is not able to effectively extract the directional features of the object. When using a general deep-learning-based object detection method, it is not suitable to locate flexible objects with arbitrary orientation. The deformable profile of flexible objects with arbitrary orientation often results in a limited proportion of small foreground and large background in the HBB.

Moreover, multi-oriented object detection uses the RBB to ensure that the foreground information is significantly larger than the background and refine redundant information from the input. However, an abrupt change in the boundary of the RBB would influence the results of the CNN backpropagation derivation calculation. It cannot guarantee that the algorithm training will converge to a certain extent and that the detection form will be error-free.

Considering the RBB boundary problem, we adopt the less problematic long-side representation and provide a novel method for finding flexible objects with arbitrarily oriented boundaries. First of all, in order to learn and represent the orientation information of flexible objects with arbitrary orientation in images, we adopt the long-side representation, which can simply and directly learn the orientation properties of objects. Then, one-stage object detection is performed in this part. The addition of the angle parameter would not influence the performance of the one-stage algorithm in training and detection time. Finally, different from the prior work, a new strategy based on using limited classification and symmetry restrictions is proposed to solve the boundary problem of the long-side representation.

## 3. Proposed Method

### 3.1. Ground Truth Generation

Ground truth (GT) refers to the labeled bounding box, which is the correct position for the object bounding box. Both the OpenCV representation and the long-side representation are suitable for label GT, but the long-side representation is more suitable for arbitrarily oriented flexible objects. OpenCV needs to solve two problems inherently, while the long-side representation only needs to solve the periodicity of the rotation angle. Therefore, we adopt the long-side representation to generate ground truth and propose a solution strategy for its periodic problem.

The ground truth is redefined, and the proportion of the foreground in the bounding boxes is increased using the long-side representation; the result of labeling is shown in Figure 1. Therefore, the model’s training data must be preprocessed. The images we labeled belong to the visual object classes (VOC) format. We extract the angle data for the long-side representation mapping and confine the angle inside $\left[0^{\circ },180^{\circ }\right)$ to fulfill the needs of training rotated bounding boxes. The parameter range of the long-side representation is $\left[-90^{\circ },90^{\circ }\right)$, which is first mapped to $\left[0^{\circ },180^{\circ }\right)$, that is, $90^{\circ }$ is added to all angle values represented by the long-side representation. The angle θ is divided into equal parts, transforming the θ parameter’s regression task into a classification task of 180 degrees rather than a numerical calculation, ensuring that the anchor adjustment does not exceed the limited scope.

The parameter form of the RBB used is [x,y,w,h,P,θ]: the *x* and *y* coordinate offsets of the prediction box’s center, the width and height of the bounding box, the confidence that an object exists, and the one-dimensional rotation angle category. The angle parameters of the RBB are used in the classification training, and the VOC format XML annotation file needs to be preprocessed. The converted format is shown in Table 1.

The center point’s coordinates, as well as the long and short sides, are normalized into the range [0,1], while the range of angle parameters is $\left[0^{\circ },180^{\circ }\right)$, with a total of 180 categories. The rounding is done during training. The R_YOLOv5 algorithm is trained using the preprocessed training set, that is, the associated JPG photographs and TXT files.

### 3.2. Network Architecture

The YOLOv5 algorithm was chosen by us as the benchmark for improvement. The YOLOv5 object detection algorithm can generate models of varying complexity using only different training parameter configurations and is well suited to devices with varying computing power.

**Figure 4:**
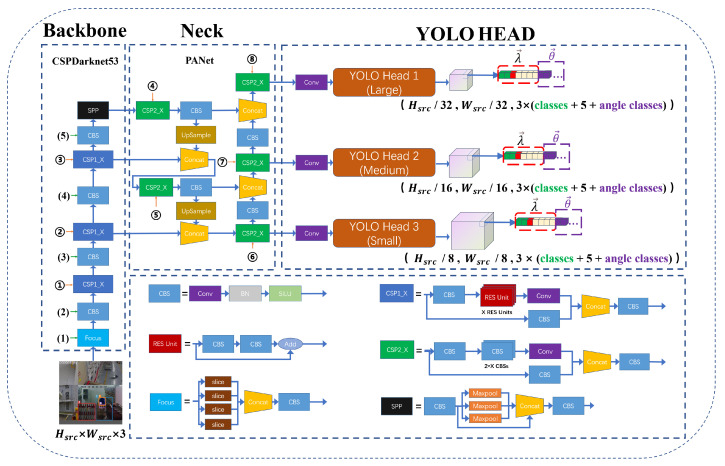
Overall structure of the proposed R_YOLOv5 network.

The network we designed with reference to the YOLOv5 structure is called R_YOLOV5. Figure 4 depicts the R_YOLOV5 network architecture, which is made up of three parts: the backbone, the neck, and the YOLO head. The backbone module is in charge of image feature extraction. The neck module is used to enhance the fusion feature information. The YOLO head module is the unit that decouples the information of objects of different sizes. The dimensions of the input image are $H_{src}\times W_{src}\times 3$, where $W_{src}$ is the width of the input image, $H_{src}$ is the height of the input image, and 3 is the number of channels of the input image.

CSPDarknet53 is used as the backbone responsible for the preliminary extraction of information from the input image, whose structure is also shown in Figure 4. The CSPDarknet53 network is mainly composed of Focus, CBS, CSP1_X, and SPP [5] modules, where CSP1_X is one of the CSP [24] structures, and the number X represents the number of superimposed modules. The details of each module can be seen in the dotted box in the lower right corner of the figure. The neck network is responsible for fusing feature information from feature maps of different sizes. Both top-down and bottom-up concatenation operations are performed on the feature data through the PANet [26]. The PANet network is mainly composed of CSP2_X, CBS, and UpSample modules, where CSP2_X is one of the CSP structures and the number X represents the number of superimposed modules. The details of each module can be seen in the dotted box in the lower right corner of the figure. The YOLO head is in charge of decoupling the feature map data. Through normal convolution, three branches are obtained. The three branches are responsible for the prediction of large-, medium-, and small-scale objects, respectively.

The special feature of the R_YOLOv5 algorithm is the YOLO head. First, the decoupling heads of the three scales need to predict the coordinate information $\vec{\lambda }$ adjusted by the anchor. The specific parameters of $\vec{\lambda }$ are (classes,P,x,y,w,h), where classes represent the probability of belonging to the category, *P* represents the probability that there is an object in this anchor, *x* and *y* represent the offset to adjust the center coordinates of the anchor, and *w* and *h* represent the scale factor for scaling and adjusting the width and height of the anchor. Then, on the basis of predicting the rectangular bounding box, the three-scale decoupling heads also predict the angle information $\vec{\theta }$ that the anchor needs to adjust. The most important thing is that the angle information is not a numerical value but a 180-dimensional angle probability.

R_YOLOv5 has four models of sizes similar to YOLOv5, and the four model sizes of R_YOLOv5 are controlled by depth control modules ①–⑧ and width control modules (1)–(5). The depth control module controls the number of channels in the model output feature, and the width control module controls the width and height of the model output feature map. The specific parameters of the depth control module are shown in Table 2, and the specific parameters of the width control module are shown in Table 3. It can be seen from Table 2 that the control of the model depth parameter is controlled by the CSP structure, which is mainly composed of two modules, CSP1_X and CSP2_X, and the number X affects the number of repetitions of the two modules at different positions. It can be seen from Table 3 that the control of the model width parameter is determined by the number of convolution kernels in the Focus and CSB modules. The more convolution kernels there are, the deeper the model can be generated. The above are the generation details of the four models R_YOLOv5s, R_YOLOv5m, R_YOLOv5l, and R_YOLOv5x.

### 3.3. Training Objective

To tackle the periodic boundary problem of long-side representation and make the objective semantics more precise, we formulate the strategies in this section. Briefly, we modified the structure of the YOLO head and added a parameter that can learn angle information, which belongs to the angle category parameter. Moreover, the category loss function is optimized to ensure that angle information can be learned.

**Figure 5:**
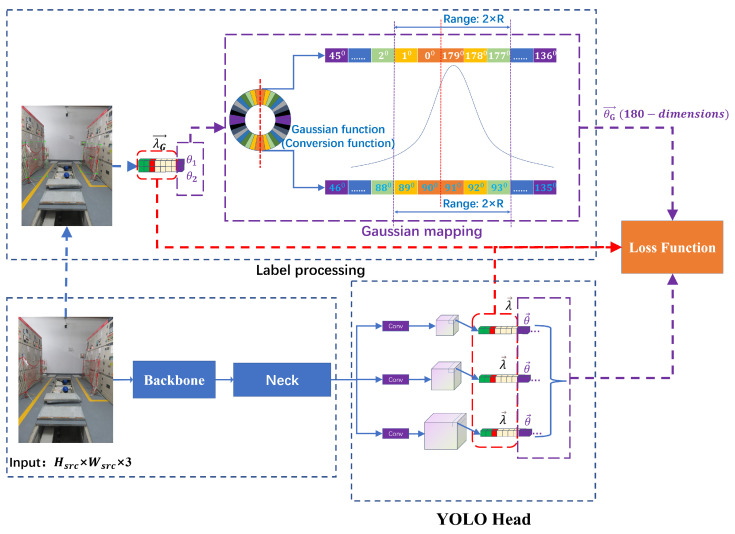
The process of training rectangular box coordinates, category confidence, class probability ($\vec{\lambda }$), and 180-dimensional category angle probability($\vec{\theta }$).

**Figure 6:**
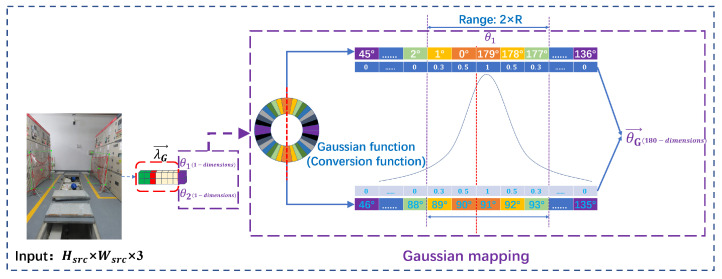
Gaussian function mapping.

Figure 5 depicts the design’s training process. The upper branch includes label processing and Gaussian function mapping, and the lower branch is the forward reasoning of the model network. The key to label processing is to process the angle value. First, the angle value is rounded up to a category, a total of 180 categories, corresponding to the angle $\theta \in [-90^{\circ },90^{\circ })$. Then, for each angle value, set the corresponding Gaussian function, and map the scalar $\theta_{1}$ and $\theta_{2}$ into a 180-dimensional Gaussian vector $\vec{\theta_{G}}$ centered on the angle scalar. The specific mapping details are shown in Figure 6. Finally, the bounding box prediction vector $\vec{\lambda }$ and the 180-dimensional angle prediction vector $\vec{\theta }$ output by the detection head module are sent to the loss calculation module, and the real label after label processing is calculated. Through the self-backpropagation of the tool framework, this is the training stage of the whole process.

The rotation angle is a periodic phenomenon in the long-side representation. There is a jump discontinuity point at $0^{\circ }$ and $179^{\circ }$, as illustrated in Figure 6, mapping adjustment. When the anchor angle is $179^{\circ }$ and the GT angle is $0^{\circ }$, this numerical jump break point will cause the anchor to fail to optimally return to the GT counterclockwise, and the anchor will return to the pointer in the way of $179^{\circ }+offset$. This condition is not beneficial to the CNN network’s derivation and backpropagation, as well as the appropriate regression that the anchor cannot achieve. The angle values of RBBs are translated into angle categories in this work, and the anchor regression parameters trained with limited angle categories are not allowed to exceed the bounds. Finally, a symmetric function constraint is used, which solves the periodic phenomenon of the angle parameters and maps the numerical distance between them to match the loss function’s calculation criteria. The algorithm’s inference speed is affected little by the YOLO head parameter increase.

The symmetry of the Gaussian function converts the angular categories with large gaps into approximately smooth probability category vectors, as shown in Figure 6. The upwardly rounded scalar angles $\theta_{1}$ and $\theta_{2}$ determine the center of the Gaussian function mapping and use this center as a Gaussian mapping function with a scope of 2R, R = 2 in the diagram. Different 180-dimensional Gaussian vector labels $\vec{\theta_{G}}$ can be generated based on different scalar angle values. The following is an example of a Gaussian map generating an angle label $\vec{\theta_{G}}$, where we represent the angle of the rotated box in long-side representation. First, the one-dimensional angle label of the GT needs to be generated according to Table 1. In the figure, the angle of the RBB is $89^{\circ }$. After being constrained by Table 1, the angle becomes $179^{\circ }$. At the same time, an array of 180 numbers in $\vec{\theta_{G}}=\left[0,\ldots ,1_{179}\right]$ is generated, and 179 is the index value of the array. Then, bringing $179^{\circ }$ into Equation (1), one can obtain the corresponding angle of $A=91^{\circ }$, update array $\vec{\theta_{G}}$, and obtain $\vec{\theta_{G}}=\left[0,\ldots ,1_{91},0,\ldots ,1_{179}\right]$. Secondly, bring 179 into Equation (2) and design the corresponding Gaussian function, and the scope of action is 2R, R=2 in Figure 6. According to this, the probability at the index 179 of the array is 1, the probability at the index 178 is 0.5, the probability at the index 177 is 0.3, and it should be noted that the operation at the index 91 of the array is consistent with this. Perform Gaussian mapping at indexes 179 and 91 to obtain the updated label array $\vec{\theta_{G}}=\left[0,\ldots ,1_{91},0.5_{92},0.3_{93},0,\ldots ,0.3_{177},0.5_{178},1_{179}\right]$ again. Finally, perform reverse addressing on the index value to obtain the final label array $\vec{\theta_{G}}=\left[0.5_{0},0.3_{1},0,\ldots ,0.3_{89},0.5_{90},1_{91},0.5_{92},0.3_{93},0,\ldots ,0.3_{177},0.5_{178},1_{179}\right]$.

(1) $$A\left(\theta \right)=\left\{\begin{array}{cc} 90^{\circ }-\theta , & \theta \in \left[0^{\circ },45^{\circ }\right]\\ \theta , & \theta \in \left[46^{\circ },135^{\circ }\right]\\ 270^{\circ }-\theta , & \theta \in \left[135^{\circ },180^{\circ }\right) \end{array}\right.$$

(2) $$\vec{\theta_{G}}=\left\{\begin{array}{cc} e^{-\frac{{\left(A-\left\lceil \theta \right\rceil \right)}^{2}}{2},}, & \theta -R<A<\theta +R\\ 0, & \;\mathrm{otherwise}\; \end{array}\right.$$

The Gaussian mapping conversion function assesses the angle error by converting the probability category of the 180-dimensional angle into the equivalent periodic value for the operation. The value of the predicted angle probability distribution of the mapping and the mapped smoothed one-hot label value can be subjected to the cross-entropy operation, which solves the boundary mutation phenomenon of the long-side representation because the symmetry of the Gaussian function is consistent with the angle symmetry of the long-side representation.

The input of the loss function changes after being converted by the Gaussian function. The loss function of the R_YOLOv5 algorithm designed in this paper is as follows. The confidence loss of the model uses the BECLogits loss function, and the equation is defined in (3):

(3) $$\begin{array}{c} {\mathrm{Loss}}_{\mathrm{conf}\;}=-\sum_{i=0}^{s^{2}}\sum_{j=0}^{B}I_{ij}^{obj}\left[{\hat{C}}_{i}^{j}\log\left(C_{i}^{j}\right)+\left(1-{\hat{C}}_{i}^{j}\right)\log\left(1-C_{i}^{j}\right)\right]\\ -\sum_{i=0}^{s^{2}}\sum_{j=0}^{B}I_{ij}^{nobj}\left[{\hat{C}}_{i}^{j}\log\left(C_{i}^{j}\right)+\left(1-{\hat{C}}_{i}^{j}\right)\log\left(1-C_{i}^{j}\right)\right] \end{array}$$

In Equation (3), *S* represents the number of grids, the feature map at each scale is divided into $S^{2}$ grids, *B* represents the number of anchor boxes generated by each grid, and $I_{ij}^{obj}$ and $I_{ij}^{nobj}$ indicate whether it is positive or negative samples. For positive samples, $I_{ij}^{obj}$ is 1 and $I_{ij}^{nobj}$ is 0, and the opposite is true for negative samples. $\hat{C_{i}^{j}}$ represents the true confidence of the sample, and the value is 0 or 1, and $C_{i}^{j}$ represents the prediction confidence of the sample.

The classification loss of this model adopts a cross entropy loss function:

(4) $$\;{\mathrm{Loss}}_{cls}=\sum_{i=0}^{s^{2}}I_{ij}^{obj}\sum_{\mathrm{CEClasses}\;}\left[{\hat{P}}_{i}^{j}\log\left(P_{i}^{j}\right)+\left(1-{\hat{P}}_{i}^{j}\right)\log\left(1-P_{i}^{j}\right)\right]$$

In Equation (4), $\hat{P_{i}^{j}}$ represents a real category, and $P_{i}^{j}$ represents the predicted category probability value.

The positioning loss of this model adopts CIoU loss:

(5) $$L_{CloU}=1-CloU=1-IoU+\frac{\rho^{2}\left(b,b^{gt}\right)}{c^{2}}+av^{2}$$

In Equation (5), ρ represents the distance between the center point of the GT box($b^{gt}$) and the center point of the prediction box(*b*) in the figure, and *c* represents the diagonal distance of the minimum closure area, which contains both the GT box and the prediction box. *a* and *v* are penalty factors used to measure the similarity of the aspect ratio.

In order to ensure that the angle classification information $\vec{\theta }$ can be trained, we optimized the classification loss function, which added the angle cross-entropy loss function.

(6) $${\mathrm{Loss}}_{\theta_{CLS}}=\sum_{i=0}^{s^{2}}I_{ij}^{obj}\sum_{C\in \;\mathrm{Classes}\;}\left[{\hat{\theta }}_{i}^{j}\log\left(P_{i}^{j}\right)+\left(1-{\hat{\theta }}_{i}^{j}\right)\log\left(1-\theta_{i}^{j}\right)\right]$$

In Equation (6), $\hat{\theta_{i}^{j}}$ indicates the real category of mapping by the conversion function, and $\hat{\theta_{i}^{j}}$ represents the predictive category of the converted function. In summary, the loss function applied to the algorithm of this article is $L=Loss_{conf}+Loss_{cls}+Loss_{CIoU}+Loss_{\theta_{CLS}}$.

### 3.4. Inference

The input RGB image outputs the prediction vector after the convolution operation of the backbone, neck, and YOLO head. As opposed to the training stage, the prediction stage does not require the reverse iteration of the loss function calculation, and the softmax processing is applied to the output of the softmax function operation on vector $\vec{\theta }$ to generate the rotating bounding box’s angle parameter.

**Figure 7:**
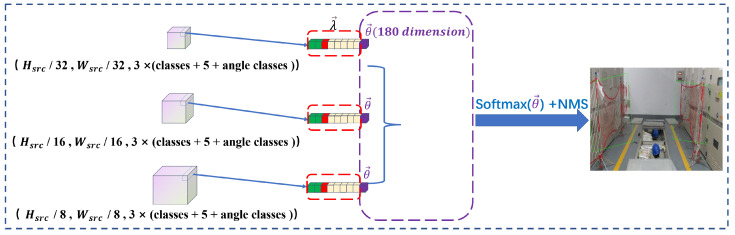
Inference process.

The inference process is shown in Figure 7. During the inference process, given the image to be detected, the forward propagation of the network will output $\left[C,P,x,y,w,h,\vec{\theta }\right]$ at the relevant position of each feature layer. Normal bounding boxes (vectors $\vec{\lambda }$) are used for the first six, and the anchor will be changed to locate the object’s approximate location. To obtain the angle value with the highest probability, apply the max operation to the 180-dimensional angle category vector. To acquire the final result, the inference output values are mapped back to the input image and non-maximum suppression (NMS) processing is performed.

## 4. Experiments

### 4.1. Datasets

First, the effectiveness of the proposed R_YOLOv5 is verified and compared with the state-of-the-art methods on the DOTA-v1.5 dataset. Then, the practicality of the proposed R_YOLOv5 is verified and compared with the original YOLOv5 on the self-built engineering power grid dataset.

DOTA [35] is the largest dataset for oriented object detection in aerial images. DOTA-v1.5 has 2806 remote sensing images taken by different satellites, together with more than 400,000 objects labeled. The classes in DOTA-v1.5 include baseball diamond (BD), tennis court (TC), ship (SH), basketball court (BC), plane (PL), ground track field (GTF), harbor (HA), bridge (BR), small vehicle (SV), soccer ball field (SBF), large vehicle (LV), helicopter (HC), swimming pool (SP), storage tank (ST), roundabout (RA), and container crane (CC). Since the annotations of the DOTA-v1.5 test set were not made public, all results in this study were evaluated on the annotated validation set. The image resolution of the DOTA dataset is too large, and a sliding window is used to segment the dataset images. The original images are cropped into 1024 × 1024 patches with a stride of 200.

The flexible object (FO) dataset was collected from the actual surveillance video of a power company. The FO dataset has 1001 images of flexible objects with arbitrary orientation captured by different surveillance spheres and cameras, together with more than 2000 objects labeled. The classes in the FO dataset include seine and fence: they are the most frequent and representative flexible objects with arbitrary orientation in the electric power scene. The FO dataset is divided by us into three parts: the training set, the validation set, and the test set. The numbers of their pictures are 742, 83, and 176, respectively, and all results in this study were evaluated on the annotated test set.

Compared with the FO dataset, the DOTA dataset has a larger amount of data and more comparable models. This dataset can be used to evaluate the effectiveness of the algorithm in this paper. The data in the FO dataset contain unique flexible objects with arbitrary orientation, and this dataset can be used to evaluate the actual effect of the algorithm in this paper.

### 4.2. Metrics

The proposed rotation-adaptive YOLOv5 model’s performance was assessed and compared to state-of-the-art models using Precision and Recall, which are commonly used metrics for detection and classification tasks. Precision represents the proportion of *k* true positive (TP) samples that are correctly judged in N samples. The equation is as follows:

(7) $$Precision=\frac{k}{N}=\frac{TP}{TP+FP}$$

Recall represents the proportion of correctly judged *k* true positive (TP) samples among the *N* samples divided by the total *M* positive samples, with the following equation:

(8) $$Recall=\frac{k}{M}=\frac{TP}{TP+FN}$$

The mAP is averaged across all AP classes. Because it takes into account the precision and recall rate of detection results, this paper uses mAP as the final evaluation index. The following is the equation for calculating it:

(9) $$mAP=\frac{\sum_{i=1}^{K}AP_{i}}{K}$$

*K* represents the object’s category, while AP is the area of the interpolated precision–recall curve and the X-axis envelope:

(10) $$AP=\sum_{i=1}^{n-1}\left(r_{i+1}-r_{i}\right)p_{\mathrm{interp}\;}\left(r_{i+1}\right)$$

Among AP, $r_{i}$ represents the recall value corresponding to the first interpolation of the precision interpolation segment in ascending order.

### 4.3. Results

Experiments were carried out to analyze and verify the proposed method for detecting flexible objects with arbitrary orientation. All the experiments were performed using a workstation that has 128 GB of RAM and an NVIDIA GTX 2080Ti GPU (11GB memory) with the CUDA 10.1 GPU acceleration library. All images in the DOTA dataset and FO dataset are scaled to 1024 × 1024 and 640 × 640 for training. For stable batch normalization and prevention of over-fitting, we set the batch size to 16 and applied an early stop method by training the networks with stochastic gradient descent for 50 epochs. The learning rate and the momentum were set to 0.01 and 0.937, respectively. The weight decay was set to 0.0005, and the confidence threshold was set to 0.25 for comparison.

#### 4.3.1. Results on DOTA-v1.5

We compare R_YOLOV5 with the FPN with Faster RCNN [9], RetinaNet [36], YOLOv4 [37], PANet [26], CDD-Net [38], SCANet [39], HTC [40], Mask R-CNN [41], and ReDet [42].

It is worth noting that the image size we input to each network is 1024 × 1024, and the comparison results are shown in Table 4. Obviously, among the methods listed in Table 4, the R_YOLOV5 series algorithm proposed in this paper has the best detection accuracy. Compared with the representative algorithm of two-stage, the FPN with the Faster RCNN [9] algorithm is the benchmark. R_YOLOV5 is generally effective at detecting small instances (e.g., HA, SP, and CC); moreover, it outperforms the previous algorithms by more than 90% when it comes to detecting large-scale variations in instances (e.g., PL and BD). In addition, in Table 4, as the number of parameters of our R_YOLOV5 increases, the accuracy gradually improves, and the algorithm achieves a better trade-off between parameters and accuracy, which further proves its efficiency.

**Figure 8:**
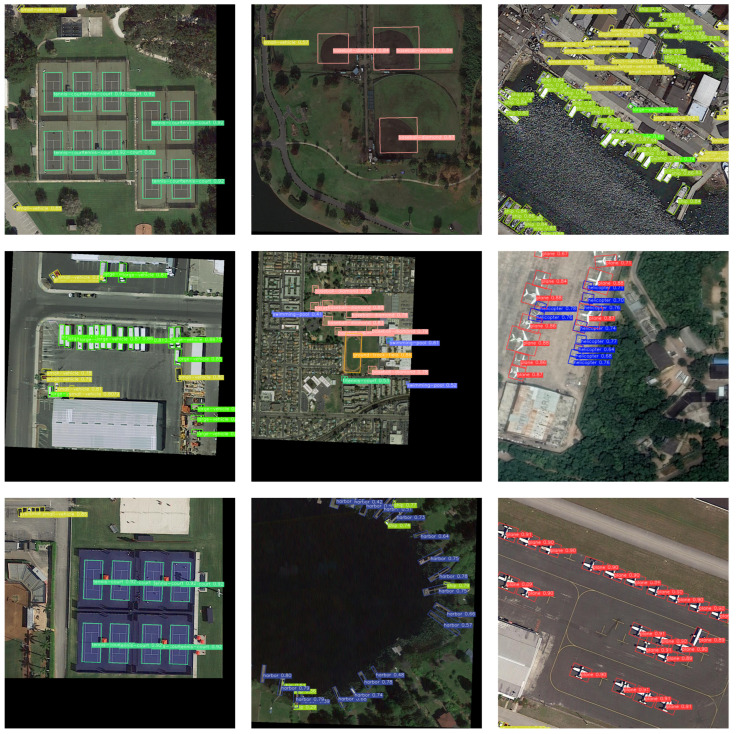
Visualization results of the R_YOLOv5 evaluation.

Compared with the representative algorithm of two-stage, the FPN with the Faster RCNN [9] algorithm is the benchmark. R_YOLOV5 is generally effective at detecting small instances (e.g., SH, SV, and PL), as shown in the third column of Figure 8. Moreover, it outperforms the previous algorithms by more than 90% when it comes to detecting large scale variations in instances (e.g., BD, TC, BC, and SBF), as shown in the first column of Figure 8. In addition, as shown in Table 4, as the size increases from R_YOLOV5s to R_YOLOV5x, the accuracy gradually improves, and the algorithm achieves a better trade-off between size and accuracy. R_YOLOv5x achieves a mAP that is about 6.84% higher than ReDet [42] on the DOTAv-1.5 dataset, and the map of R_YOLOV5x reached 74.5%, which further proves its efficiency.

However, the R_YOLOv5 algorithm also has some classes with low detection accuracy, such as GTF, BR, HC, SP, RA, and CC, especially the CC, HC, and SP classes. CC is a newly added object to be detected in the DOTA-v1.5 dataset. This object has fewer data and more interference. The best map of the R_YOLOv5 algorithm is only 23.90%; The BR objects in the DOTA-v1.5 data are mostly distributed in long strips, which are easy to confuse with the ground in the overhead view, which is not conducive to being detected. The best map of the R_YOLOv5 algorithm is only 60.20%; The situation of RA objects is similar to that of BR objects, both belonging to the infrastructure of the road, and it is not easy to distinguish them from the overhead perspective. The best map of the R_YOLOv5 algorithm is only 63.40%.

Figure 8 displays a portion of the visual results of R_YOLOV5 on the DOTA dataset. The displayed findings contain the category name and the probability that the object belongs to that category. The detected objects in the picture are represented by revolving boxes of various colors. This can be seen in Figure 8. Whether it is a large object (BD) or a small object (SV), the R_YOLOv5 algorithm can achieve good detection, and the ability of the algorithm to capture object direction information has been verified. In particular, the R_YOLOv5 algorithm is suitable for detecting large and narrow objects, such as TC and LV. The detection effect in Figure 1 and the mAP values of 97.00% and 84.6% in Table 4 have verified the effectiveness of the method. In summary, the results on the DOTA-v1.5 dataset show that the R_YOLOv5 algorithm can effectively capture directional objects and is suitable for detecting large and narrow objects with arbitrary orientation.

Furthermore, we conducted a targeted comparison between YOLOv5 and R_YOLOv5 on the FO dataset. The seines and fences at the power grid maintenance and inspection site have the characteristics of large objects and large scale spans. The next section is the performance test results of R_YOLOV5 for this specific application scenario.

#### 4.3.2. Results on FO Dataset

**Figure 9:**
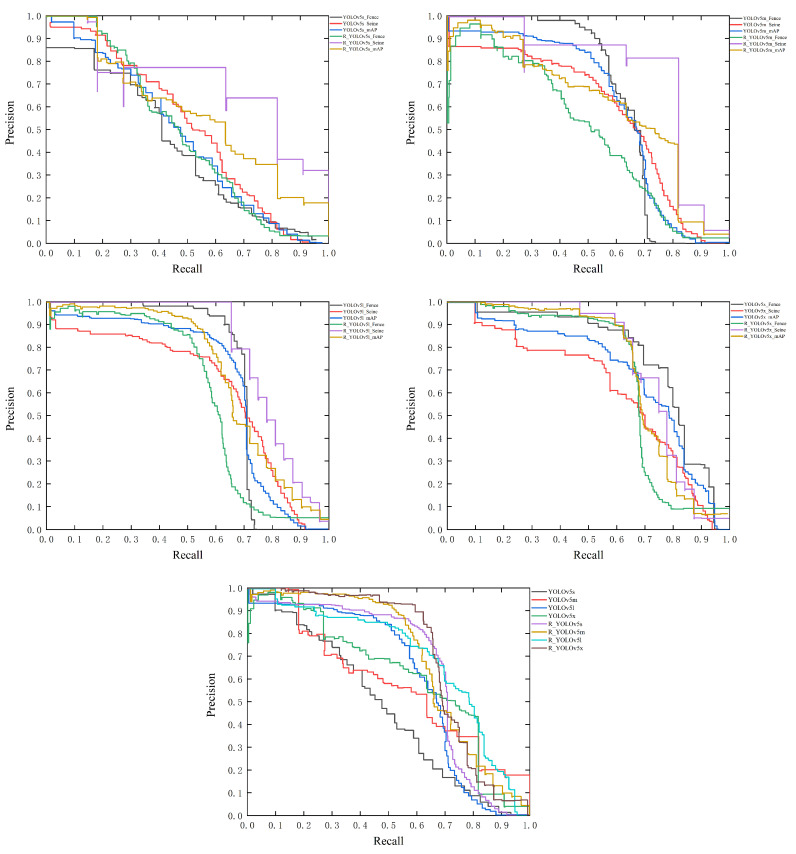
Comparison of PR curves between R_YOLOv5 and YOLOv5 on the FO dataset.

The FO dataset is collected and labeled under the monitoring of electric power scenes and contains a large number of flexible objects with arbitrary orientation, such as seines and fences. In order to compare the detection gap between the R_YOLOv5 and the original YOLOv5 in detecting flexible objects with arbitrary orientation and to test the practical application performance of the R_YOLOv5 algorithm, we use the FO dataset to evaluate the above two algorithms. The experimental results of the FO dataset are shown in Table 5 and Figure 9.

In the YOLOv5 series of algorithms, YOLOv5s is the lightest model. The detection accuracy is not ideal; the mAP is only 47.7%. In our R_YOLOv5s model, the mAP has substantially improved, reaching 57.9%.

The mAP gap between the YOLOv5m model and our R_YOLOv5m model is small; the experimental data reveal that the seine’s mAP is significantly better than the original YOLOv5m model.

The YOLOv5l model’s parameters already fall into the large model. The AP of the seine and fence has substantially improved as compared to the YOLOv5m model, and the improvement effect of our R_YOLOv5m model is more obvious, with the AP of the seine reaching 79.4%.

The YOLOv5x model is the largest of the YOLOv5 models. The detection accuracy improves when the model weight parameters are increased. The mAP has risen to almost 70%. Our revised R_YOLOv5x model is around 2% more accurate than the YOLOv5x model, with a significantly better AP for detecting seine objects than the YOLOv5x model.

The YOLO series algorithm is a one-stage object detection algorithm, and its algorithm is real-time (20 fps). It can be seen from Table 5 that the original YOLOv5 model can basically achieve real-time detection, except for the YOLOv5x model. Compared with the original YOLOv5 model, the R_YOLOv5 model has a slight decrease in detection speed, from the s-series model to the x-series model by 2.2 fps, 2.2 fps, 1.5 fps, and 1.1 fps, respectively. The increased angle learning parameters of the R_YOLOv5 model lead to a decrease in model reasoning speed, but the reduction in fps in R_YOLOv5 detection speed is acceptable.

The mAP of the YOLOv5 model increases as the depth and width of the model increase, as seen in Figure 9. When we look at the same set of models before and after improvement, we can see that the improved model’s mAP is higher than the original YOLOv5 model, demonstrating that our strategy is more targeted and effective in detecting flexible objects with arbitrary orientation.

**Figure 10:**
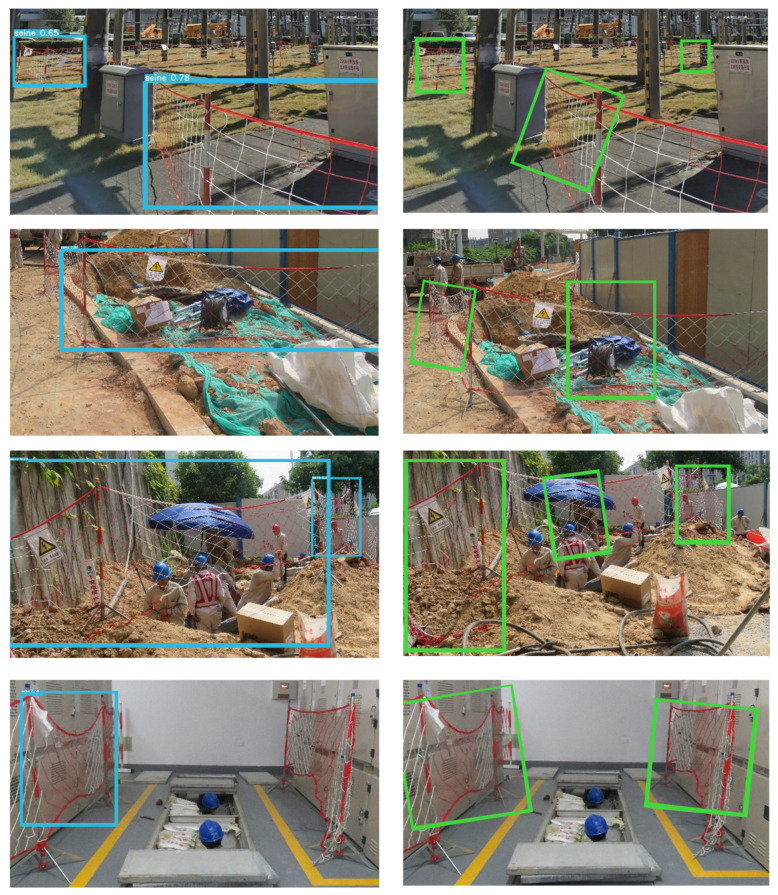
Comparison of detection results before and after YOLOv5 changes. The blue rectangle on the left is the detection effect of HBB, and the green rectangle on the right is the detection effect of RBB. It shows that the improved RBB not only can detect the seine accurately but also can provide the orientation information of the seine.

Figure 10 shows a comparison of the detection impact of YOLOv5 with the detection result of our algorithm. The YOLOv5 algorithm suffers from missing detection, the inability to precisely detect the object, and the inability to determine the object’s direction. The algorithm in this paper performs better on flexible objects with arbitrarily oriented detection, allowing it not only to detect the object more accurately but also to offer objective direction information.

## 5. Conclusions

In this paper, we proposed a new rotation-adaptive YOLOv5 object detection method suitable for detecting flexible objects with arbitrary orientation in power monitoring scenarios. Flexible objects with arbitrary orientation are difficult to detect due to the span being large and easy to deform, and in the annotations labeled by the HBB, it occupies a small proportion, which is the problem of foreground and background ratio imbalance. A new detection network, R_YOLOv5, was designed by using the long-side representation and adding angle parameter learning parameters into YOLOv5. Moreover, we did label preprocessing, optimized the loss function, and set the Gaussian mapping constraint function operations. Combining these techniques, our proposed method, which belongs to a one-stage algorithm, achieved excellent performance on the DOTAv-1.5 and FO datasets while remaining computationally efficient for real-time detection of flexible objects with arbitrary orientation tasks. The DOTAv-1.5 dataset experiments show that the R_YOLOv5 algorithm is the algorithm with the highest mAP in the table. The mAP of R_YOLOv5x can reach 74.5%, which is 6.84% higher than ReDet. The FO dataset experiments show that the algorithm not only can detect in real time but also can accurately detect flexible objects with arbitrary orientation and provide direction information, which solves the problem of low detection accuracy of flexible objects with arbitrary orientation in power monitoring scenarios. On the FO dataset, the mAP of R_YOLOv5x can reach up to 71.25%, and the mAP of all series is higher than that of the original YOLOv5. The runtime of R_YOLOv5 is a little lower than the original YOLOv5, but it is acceptable. In future work, we will consider the method based on instance segmentation that uses the rotating bounding box to more perfectly detect flexible objects with arbitrary orientation. This method does not belong to real-time detection. It focuses more on detection accuracy and requires more powerful hardware support.

## Figures and Tables

**Table 1:** Conversion Format and Scope Limits for Annotations.

Category	Center X	Center Y	Long Side	Short Side	Angle
0 (seine)	0–1	0–1	0–1	0–1	$\left[0^{\circ },180^{\circ }\right)$
1 (fence)	0–1	0–1	0–1	0–1	$\left[0^{\circ },180^{\circ }\right)$

**Table 2:** Depth control parameters used from ① to ⑧.

CSP Structure	R_YOLOv5s	R_YOLOv5m	R_YOLOv5l	R_YOLOv5x
①	CSP1_1	CSP1_2	CSP1_3	CSP1_4
②	CSP1_3	CSP1_6	CSP1_9	CSP1_12
③	CSP1_3	CSP1_6	CSP1_9	CSP1_12
④	CSP2_1	CSP2_2	CSP2_3	CSP2_4
⑤	CSP2_1	CSP2_2	CSP2_3	CSP2_4
⑥	CSP2_1	CSP2_2	CSP2_3	CSP2_4
⑦	CSP2_1	CSP2_2	CSP2_3	CSP2_4
⑧	CSP2_1	CSP2_2	CSP2_3	CSP2_4

**Table 3:** Width control parameters used from (1) to (5).

Convolution Kernels Number	R_YOLOv5s	R_YOLOv5m	R_YOLOv5l	R_YOLOv5x
(1)	32	48	64	80
(2)	64	96	128	160
(3)	128	196	256	320
(4)	256	384	512	640
(5)	512	768	1024	1280

**Table 4:** Performance comparisons on DOTA-v1.5 validation set. The numbers in bold in the table are the highest AP values for this object.

Methods	Detector	mAP/%	BD	TC	SH	BC	PL	GTF	HA	BR	SV	SBF	LV	HC	SP	ST	RA	CC
FPN with Faster RCNN [9]	HBB	57.30	70.10	86.00	40.30	69.40	78.60	68.50	59.50	55.10	23.70	61.10	45.40	68.30	64.50	46.40	56.20	24.40
RetinaNet [36]	HBB	33.50	44.50	75.10	33.40	30.80	76.00	32.50	35.80	32.60	10.70	13.00	33.30	0.20	43.90	31.20	42.40	0.00
YOLOv4 [37]	HBB	55.60	61.70	88.30	79.50	55.60	85.20	35.20	69.80	32.60	37.00	34.40	64.00	67.60	58.50	64.80	54.20	0.70
PANet [26]	HBB	61.20	74.10	89.60	58.40	67.00	85.90	64.50	67.90	51.50	27.70	63.40	56.20	71.30	73.40	61.30	59.20	7.60
CDD-Net [38]	HBB	61.30	74.70	89.80	49.20	71.40	81.40	70.10	69.90	55.30	25.30	65.60	51.50	71.30	60.40	53.30	58.20	32.70
SCANet [39]	HBB	64.00	77.20	90.30	53.70	73.20	81.10	**72.50**	70.50	**62.40**	25.60	65.30	52.70	**77.60**	68.80	52.80	63.50	**36.70**
HTC [40]	RBB	64.47	74.41	90.34	79.89	75.17	78.41	63.17	72.13	53.41	52.45	48.44	63.56	56.42	74.02	67.64	69.94	12.14
Mask R-CNN [41]	RBB	64.54	77.41	90.31	79.74	74.28	78.36	56.94	70.77	53.36	52.17	45.49	63.60	61.49	73.87	66.41	71.32	17.11
ReDet [42]	RBB	67.66	82.63	90.83	87.82	75.81	79.51	69.82	75.57	53.81	52.76	49.11	75.64	58.29	**75.17**	68.78	**71.65**	15.36
**R_YOLOv5s**	RBB	71.20	82.00	96.10	93.30	77.10	96.40	66.90	83.00	53.30	67.80	58.40	82.00	67.30	67.90	70.50	56.50	20.10
**R_YOLOv5m**	RBB	73.10	84.20	96.20	94.90	80.00	97.00	62.80	85.20	56.00	70.20	61.40	83.60	73.40	71.70	74.70	59.90	17.70
**R_YOLOv5l**	RBB	73.60	86.00	96.50	94.90	**83.40**	97.40	69.20	84.70	56.00	70.70	**66.60**	84.10	76.50	71.50	70.40	63.40	5.60
**R_YOLOv5x**	RBB	**74.50**	**86.00**	**97.00**	**96.00**	83.10	**97.60**	66.70	**86.80**	60.20	**74.30**	62.80	**84.60**	67.30	70.10	**78.60**	57.70	23.90

**Table 5:** Average precision and mAP on the FO dataset before and after YOLOv5 changes.

Method	Object	AP/%	mAP/%	Recall/%	Precision/%	Time/ms	FPS
YOLOv5s	Seine	51.60	47.70	50.70	74.50	22.68	44.10
	Fence	43.80		39.20	89.80		
R_YOLOv5s	Seine	67.72	57.90	57.70	70.20	23.87	41.90
	Fence	48.08		42.00	93.30		
YOLOv5m	Seine	57.60	60.70	55.30	62.80	35.21	28.40
	Fence	63.70		76.00	86.90		
R_YOLOv5m	Seine	75.70	62.90	53.50	71.70	38.17	26.20
	Fence	48.90		75.50	85.30		
YOLOv5l	Seine	62.20	65.50	70.50	69.90	42.02	23.80
	Fence	68.90		77.10	85.70		
R_YOLOv5l	Seine	79.40	68.90	80.00	80.10	44.84	22.30
	Fence	58.40		76.20	84.90		
YOLOv5x	Seine	62.00	69.60	80.50	74.50	51.02	19.60
	Fence	77.30		87.20	85.80		
R_YOLOv5x	Seine	74.80	71.25	80.30	74.10	54.05	18.50
	Fence	67.70		76.90	74.80		

## Data Availability

The data presented in this study are available on request from the corresponding author.
